# Validation of a novel, low-fidelity virtual reality simulator and an artificial intelligence assessment approach for peg transfer laparoscopic training

**DOI:** 10.1038/s41598-024-67435-6

**Published:** 2024-07-19

**Authors:** Peter Zoltan Bogar, Mark Virag, Matyas Bene, Peter Hardi, Andras Matuz, Adam Tibor Schlegl, Luca Toth, Ferenc Molnar, Balint Nagy, Szilard Rendeki, Krisztina Berner-Juhos, Andrea Ferencz, Krisztina Fischer, Peter Maroti

**Affiliations:** 1 3D Printing and Visualisation Centre, University of Pecs, Medical School, Boszorkany Str. 2, Pecs, 7624 Hungary; 2 Department of Public Health Medicine, University of Pecs, Szigeti Str. 12, Pecs, 7624 Hungary; 3 Medical Skills Education and Innovation Centre, Medical School, University of Pecs, Szigeti Str. 12, Pecs, 7624 Hungary; 4 Department of Surgery and Vascular Surgery, Tolna County Janos Balassa Hospital, Beri Balogh Adam str. 5-7, Szekszard, 7100 Hungary; 5 Department of Behavioural Sciences, Medical School, University of Pecs, Szigeti Str. 12, Pecs, 7624 Hungary; 6 Szentágothai Research Centre, University of Pecs, Pecs, Ifjusag str. 20., 7624 Hungary; 7 Department of Orthopaedics, Medical School, University of Pecs, Akac Str. 1, Pecs, 7632 Hungary; 8 Department of Neurosurgery, Medical School, University of Pecs, 2 Ret Street, Pecs, 7624 Hungary; 9 Department of Surgical Research and Techniques, Heart and Vascular Centre, Semmelweis University, Nagyvarad Square 4, Budapest, 1089 Hungary; 10 Department of Radiology, Brigham and Women’s Hospital, Harvard Medical School, 75 Francis St, Boston, MA 02115 USA

**Keywords:** Biomedical engineering, Health care, Medical research, Mathematics and computing

## Abstract

Simulators are widely used in medical education, but objective and automatic assessment is not feasible with low-fidelity simulators, which can be solved with artificial intelligence (AI) and virtual reality (VR) solutions. The effectiveness of a custom-made VR simulator and an AI-based evaluator of a laparoscopic peg transfer exercise was investigated. Sixty medical students were involved in a single-blinded randomised controlled study to compare the VR simulator with the traditional box trainer. A total of 240 peg transfer exercises from the Fundamentals of Laparoscopic Surgery programme were analysed. The experts and AI-based software used the same criteria for evaluation. The algorithm detected pitfalls and measured exercise duration. Skill improvement showed no significant difference between the VR and control groups. The AI-based evaluator exhibited 95% agreement with the manual assessment. The average difference between the exercise durations measured by the two evaluation methods was 2.61 s. The duration of the algorithmic assessment was 59.47 s faster than the manual assessment. The VR simulator was an effective alternative practice compared with the training box simulator. The AI-based evaluation produced similar results compared with the manual assessment, and it could significantly reduce the evaluation time. AI and VR could improve the effectiveness of basic laparoscopic training.

## Introduction

Simulation-based education has proven to be effective in surgical education, complementing the earlier traditional Halstedian apprenticeship model^1–3^. The knowledge gained in a simulation environment can be transferred to surgical practice, as several studies have shown positive results in laparoscopic camera navigation, significant improvement in video eye–hand skills and better performance of many procedures after simulation-based training, such as colonoscopy or endoscopic sinus surgery^4–7^.

The teaching of basic surgical skills is a compulsory part of medical education worldwide, in which training in laparoscopic skills is crucial. This can be achieved in many different forms due to the emergence of medical simulation. From the simplest, basic task trainers, such as suturing pads and box trainers, to complex, high-fidelity and computer-based simulators, various solutions are available to students and trainers^8^.

High-fidelity simulators can be used to perform both basic and specific complex simulations and exercises. These are effective tools for surgical education, but the devices and software components are expensive and mainly available in training centres; thus, their availability is limited compared with low-fidelity simulators^8–10^. Furthermore, due to the COVID-19 pandemic, there has been an increased demand for distance learning solutions, even in traditional presence-based surgical education, in which simple and cost-effective simulators are required^11,12^. It should be emphasised that distance education methods in surgical education have been proven to be effective and favoured approach by teachers and students^13,14^.

To maximise the effect of traditional or simulation-based training strategies, the implementation of standardised training protocols followed by internationally uniform assessments, such as scoring systems, is fundamental and extensively available^15–17^.

The Fundamentals of Laparoscopic Surgery (FLS) was developed by the Society of American Gastrointestinal and Endoscopic Surgeons and has become one of the most popular and widely used laparoscopic training programmes worldwide^18–20^. FLS has five simulation exercises in a box trainer, and it has been proven efficient for learning laparoscopic skills by both graduate and postgraduate students^18–20^. Although basic trainers are effective in simulation-based training, they have the major disadvantage of requiring objective evaluation and standardisation, which is still often unavailable^21^. This issue also applies to box trainers, as in many cases, instructors subjectively evaluate the performed tasks (e.g. peg transfer, stitches, general instrument manipulation) onsite visually or offline by evaluating the video recording. Aside from being subjective, these traditional evaluation methods require significant time investment and demands on the capacity of instructors^21^.

To overcome the difficulties in traditional evaluation methods, there is extensive ongoing research for implementing novel evaluation methods, such as image-processing algorithms and artificial intelligence (AI), for objective evaluation in surgical education^21–25^. The literature is diverse regarding the procedures used and the practices studied in AI-based assessments. Based on the reviews, one of the most important challenges is the highly limited availability of published datasets suitable for machine learning. Therefore, the majority of studies used the same datasets, such as the JHU-ISI Gesture and Skill Assessment Working Set (JIGSAWS) or Cholec80^26,27^. JIGSAWS is a robotic surgical dataset containing three basic surgical tasks, namely suturing, needle passing and knot-tying, while Cholec80 consists of 80 videos of laparoscopic cholecystectomy performed by 13 experts^26,27^. For task evaluation and analysis, kinematic data measured using sensors or image processing can be used^21^. However, it should be noted that most of the publications did not go beyond a comparison of manual and automatic analyses. Thus, the important features of practitioners’ skill development and the effects of the novel assessment were not evaluated^21–24^.

A considerable improvement in AI-based evaluating systems was presented by Yamazaki et al. by training an AI model to detect 10 different surgical instruments from laparoscopic gastrectomy videos and generated heatmaps, which helped surgical experts determine the type of surgery performed^28^. Using a plain evaluation method, Belmar et al. performed an automatic object detection for the peg transfer exercise, enabling the objective measurement and evaluation of the required time to fulfil the exercise, but no investigation of the pitfalls (e.g. missing handover) was conducted^29^. More detailed AI-based evaluation methods were developed, such as pitfall recognition or the effect of force feedback on the FLS task success rate. However, these approaches require significant and often complex modifications of the basic FLS trainers, which could be a major limitation in accessibility^30,31^.

As a novel approach, laparoscopic training in immersive virtual reality (VR) has been available for surgical skill training in recent decades, and it has been proven to be a useful tool for improving surgical skills and has been found to be equally effective as traditional simulation laparoscopic training^28^. VR-based simulators have also been shown to be useful in determining surgical skill level^32^. Qin et al. compared self-developed VR, augmented reality (AR), cognitive virtual reality (CVR) and mixed reality (MR) simulators with a box trainer based on questionnaires completed by 32 practitioners^33^. The results showed that the traditional box trainer was the most favoured in terms of haptic sensation, while the box trainer and the MR simulator provided the most realistic visual appearance^33^. Although data including exercise time, distance travelled by the devices and the number of drops were analysed and measured on the simulators, an objective analysis of the performance was not performed on the box trainer^33^.

Based on international scientific literature, access to high-fidelity simulators is limited for simulation-based surgical education, whereas low-fidelity simulators are widely available. However, it should be noted that an objective and automatic evaluation is not accessible in the vast majority of cases^8–10^. Considering current knowledge, AI is suitable for developing an objective assessment of existing and new simulators. VR solutions could be implemented in newly developed simulators with automatic assessments, enabling the extraction of accurate spatial data from the simulator during the exercise. These two cutting-edge technologies (AI and VR) are promising and are rapidly developing areas of the modern simulation-based training field due to the growing demand for distance learning and data-driven personalised education^21,34^.

Although previous research results have shown the potential of immersive VR in simulation-based training, there is a strong debate about whether the solution is capable of replacing the current solutions^34^. The goal of the present study is to develop and validate a system that is capable of objective evaluation and that can significantly reduce the assessment time of basic surgical skills, aiming to improve the efficacy of the training and evaluation process. As a first step, the goal is to design, develop and validate an immersive VR simulator that offers training opportunities that are at least as effective as the traditional box trainers for the peg transfer test. Following the current digitalisation trends and ‘data-driven education’, this study does not restrict the scope to VR environment development but also aims to automate the assessment of the current low-fidelity simulator with an AI-based algorithm. To the best of our knowledge, this study is the first to examine the usability of VR and AI technologies in graduate-level medical education using an automated assessment method that measures the participants’ performance in traditional box training and in a virtual environment. The performance and attitude of a relatively high number of students were explored during the procedure.

The presented algorithm attempts to evaluate the standardised peg transfer exercise of the FLS programme based on AI object, pitfall and error detection in onsite box trainers and in a VR environment. Through this, the system aims to support the improvement of simulation-based medical education by incorporating innovative technologies to create a data-driven, automated, objective assessment in the field of surgical education.

## Materials and methods

Based on the current regulations, the Regional Ethics Committee of the University of Pecs Medical School certified the research to be conducted without ethical authorisation (9749-PTE 2023). Informed consent was obtained from all subjects. All methods were carried out in accordance with relevant regulations. All data were collected anonymously, and the study was conducted in accordance with the Helsinki Declaration.

To objectively measure the training ability of the novel, self-developed, low-fidelity VR simulator in basic laparoscopic skills, the peg transfer test was used in the simulator and in a conventional FLS All-In-One Trainer System (PN: 50306 Limbs & Things inc., Savannah, GA, USA) as a control. Furthermore, to objectively validate the novel, automatic, AI-based assessment software, the peg transfer test results were evaluated by the software, and as a control with the standard, manual evaluation process were compared.

### The development of a peg transfer VR simulator

**Figure 1:**
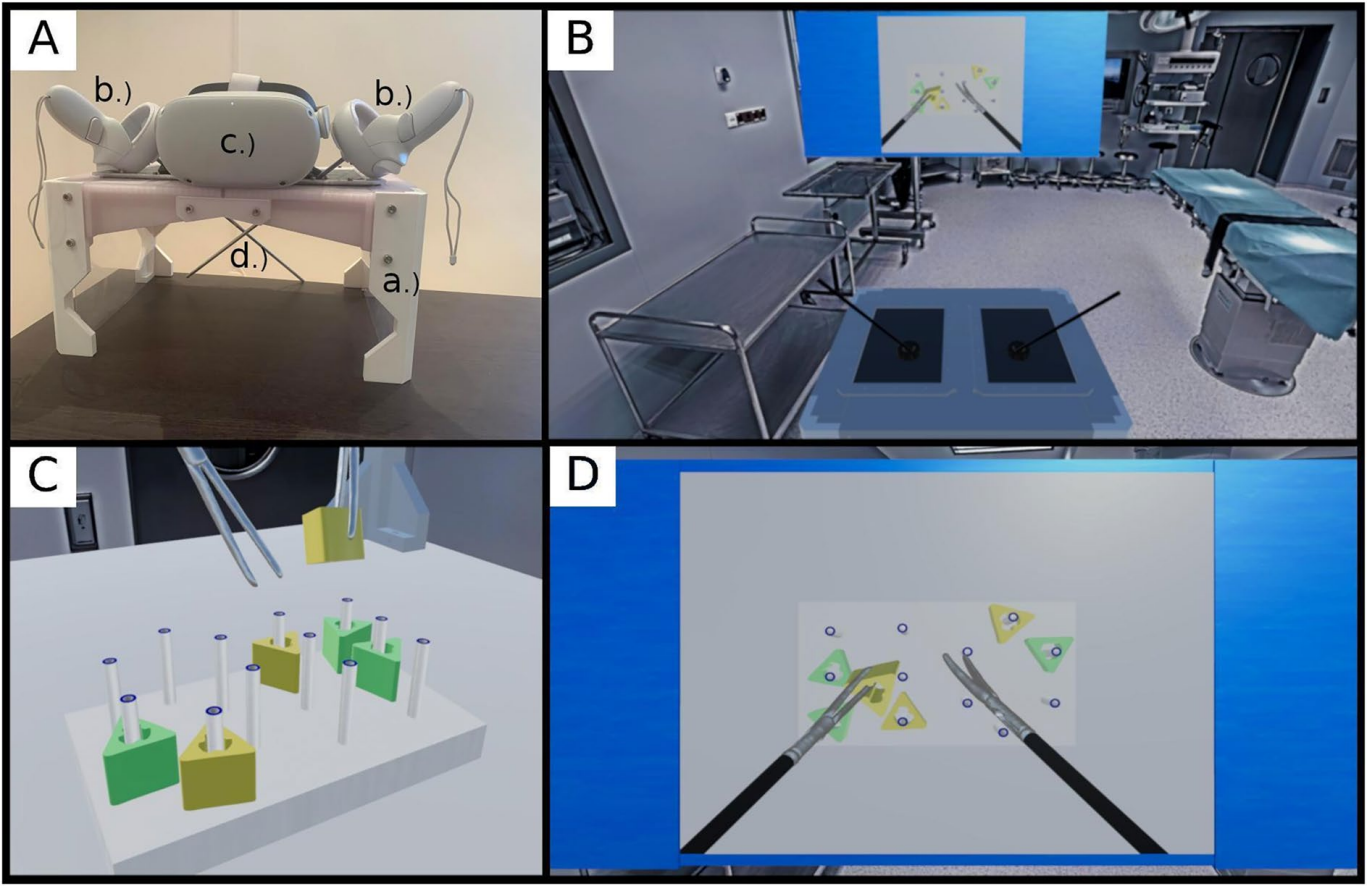
VR simulator hardware and the playground for the peg transfer exercise. Panel (**A**): The pelvic trainer used for the simulation of the FLS peg transfer task. Part a.) represents the box created with a 3D-printing technique. Part b.) indicates the 3D-printed grippers attached to the VR controllers. Part c.) is the Oculus Quest 2 VR goggles and part d.) denotes the metal rods used for the simulation of laparoscopic devices. Panel (**B**): Virtual operation theatre used in the simulation. Panel (**C**): FLS peg transfer tasks, as seen in the VR goggles (side view). Panel (**D**): FLS peg transfer tasks, as seen in the VR goggles (top view). The models and images are the sole property of the University of Pecs.

The VR simulator used in the course was a proprietary development that ran on Meta Quest 2 (Facebook Technologies, LLC, Menlo Park, CA, 94,025, USA). The Unity framework (Unity Technologies, 30 3rd Street, San Francisco, CA, USA) was used for surgical simulation software development, similar to previous studies^35,36^. For the VR simulator, the objects of the traditional FLS trainer (rubber ring, dissector and peg board) were three-dimensional (3D) models created by our research team using a maximum of 20,000 polygons per model and a maximum of 4 k resolution for texture. As recommended by the framework documentation, the interactions of the objects in the VR simulation were implemented using Unity’s physics system. Each object in the simulation had a mesh with a mesh collider and a rigid body used to set the weight and constraints on the external forces. The physics of the system implemented mesh colliders to detect collisions and overlap of objects and used rigid body information to determine which object would be repealed. Based on the collision data, the software could calculate critical events of the simulation, such as grabs, collisions or drops. To make the user experience as realistic as possible, additional 3D-printed hardware elements, such as grasps for the VR controller, a metal rod representing the laparoscopic device and a pelvic trainer box, were added to the system. Figure 1 shows the hardware elements of the simulator and the peg transfer playground. The complete list of the parts used and the blueprint of the simulator can be found in the data repository.

### VR simulation testing protocol

The course protocol had three parts, starting with initial testing (pre-course test) of FLS-naive students on the conventional trainer. The students were randomised using the RANDBETWEEN function of Microsoft Excel (v16.0.16502.42308 Microsoft Corp., Redmond, WA, USA) into the VR simulator group or control group (traditional trainer) to perform the practice training. In the second part of the protocol, the students were instructed to participate in the practice training, which consisted of the peg transfer exercise performed four times in two weeks during the preset dates either in the VR simulator or in the conventional trainer based on the randomisation. In the third part of the protocol, the students were instructed to perform the peg transfer exercise in the traditional FLS trainer (post-course test). For both the pre- and post-course tests, the students had two attempts, and the better results were considered to avoid unintentional mistakes irrespective of technical skills. The test protocol was established in agreement with the University of Pecs Medical School’s graduate surgical training plan.

The testing was performed on 65 third-year medical students as an extension of the ‘Basics of Surgery’ obligatory course. For the participants, the only exclusion criterion was any type of previous laparoscopic experience. The mentor during the training protocol was a surgeon with more than 10 years of experience in both theoretical and practical surgical education, operatively aided by two skilled technicians.

Throughout the training protocol, the practice and the testing exercise were the same regardless of the VR or conventional training device—that is, to accomplish the peg transfer exercise performed using two Maryland dissectors according to the FLS specifications. Each object had to be lifted from the peg, transferred to the other clamp in mid-air without using the board or pegs for assistance and then placed on any of the empty pegs on the other side of the pegboard. This procedure had to be repeated in another direction. Five students were excluded because they either did not attend the post-course test or only partly participated in the practice session.

Following the practice period, the VR group participants were asked to complete a five-point Likert scale questionnaire about their subjective impressions of the VR simulator and what they thought of it compared with the conventional FLS trainer. The questionnaire was created using Google Forms (Google LLC, Amphitheatre Parkway, Mountain View, CA, USA), and it was administered anonymously.

**Figure 2:**
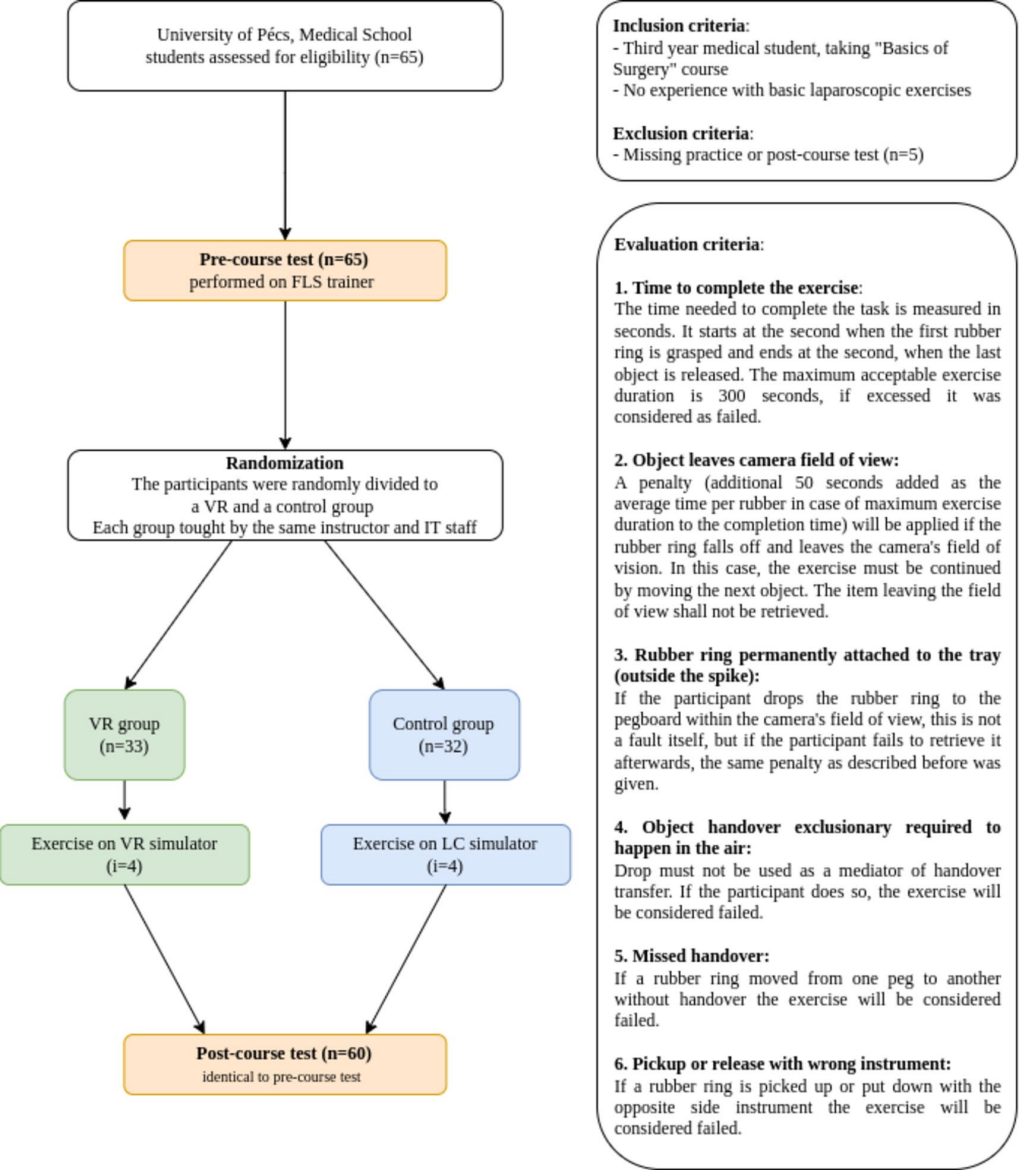
The protocol followed in the study. n: number of participants; i: number of attempts the task has been completed during practice by each participant.

The pre- and post-course tests were recorded, and the videos were evaluated by six professionals who had more than three years of experience in surgical education. Each video was evaluated by two professionals. If there was any incoherence between the two evaluators, a third professional was brought in, and all three evaluators decided on the questionable event or pitfall. The evaluation of the pre- and post-course tests was based on the assessment criteria of the FLS peg transfer test (details in Fig. 2).

### Development of AI-based automatic assessment software

To establish an automatic assessment system, AI-based software was developed with the same evaluation criteria used in a standard assessment method (Fig. 2). During the automatic evaluation, the algorithm works frame by frame through the exercise video, using the trained AI models to detect relevant objects and determine their position on the image. Based on this data and the information gained, the software analyses the execution of the exercise and determines the result. The evaluation is automated, objective and reproducible.

During the peg transfer exercise, the objects that had to be detected in the video were the pegs, the Maryland dissectors and the rubber rings, which had to be transferred, while the event to be detected was the grab (when one Maryland dissector grabs a rubber ring). Henceforth, they are referred to as the objects of the study.

A YOLOv8 AI model was trained using supervised learning with a dataset consisting of 22,675 images for training/validation with a split of 12.5%, and 24,628 images were used for testing in the PyTorch framework^37,38^. The optimal number of epochs was determined based on the learning curves, initially using the 300 epochs proposed in the YOLOv8 documentation. For our custom training dataset, the optimal number of epochs was 100. To generate the training dataset, videos of the training sessions during the course were used. The exercises were randomly selected, and the recordings were split into frames on which the necessary annotation preparation was performed using CVAT software^39^. On the online CVAT interface, the annotator manually annotates the images with bonding boxes for the seen objects. The datasets can be exported in the appropriate format from the online interface. The following training datasets were used: 14,223 annotated images for grab, 1790 annotated images for dissectors and rubbers and 4142 annotated images for pegs. The validation dataset consisted of 1778 annotated images for grabs, 224 annotated images for dissectors and rubbers and 518 annotated images for pegs. To evaluate the models, the following test datasets were used: 6532 annotated images for grab, 11,195 annotated images for dissectors and rubbers and 6901 annotated images for pegs.

**Figure 3:**
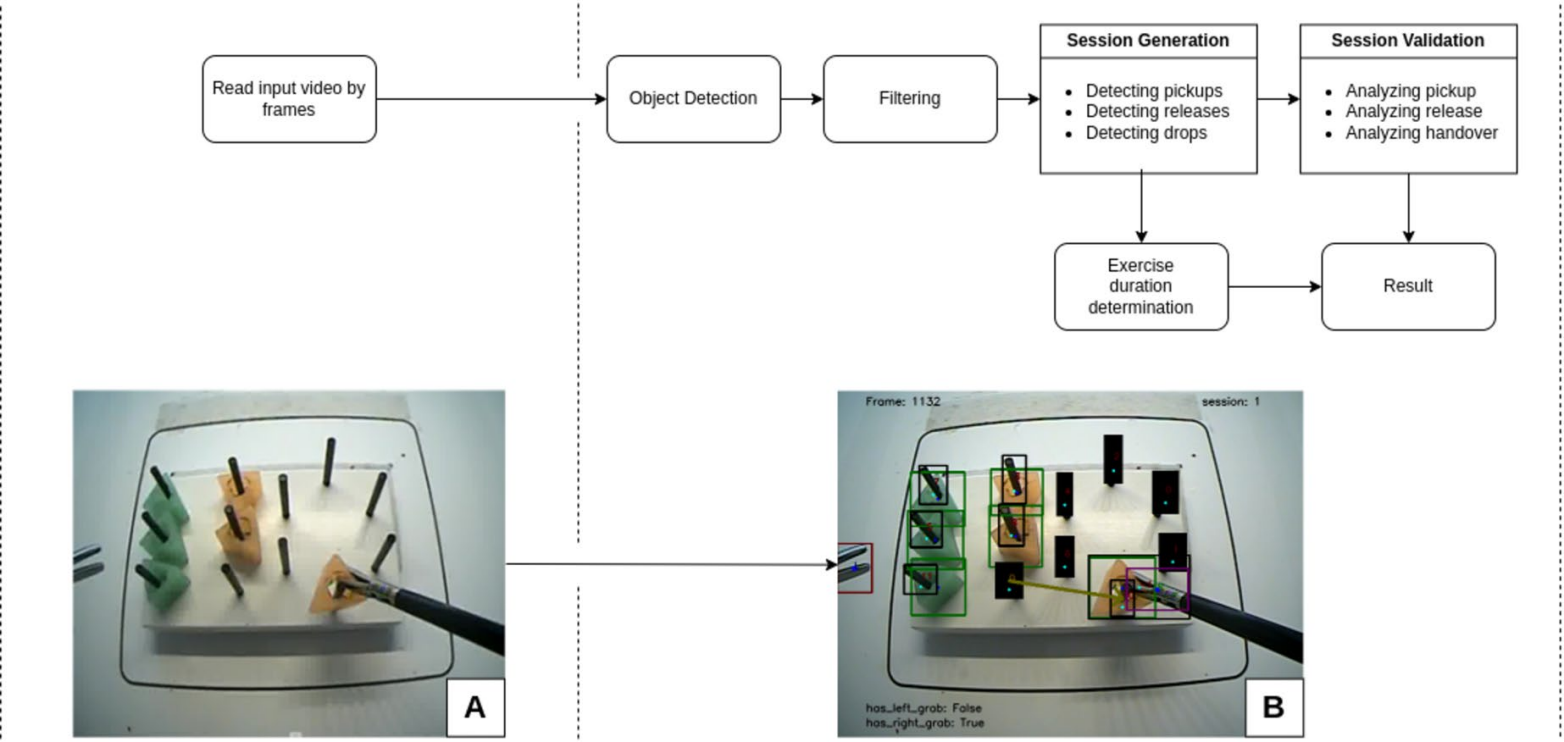
Representation of the developed AI-based evaluation algorithm. Panel (**A**): Input image Panel (**B**): Visualisation of exercise validation (current session and objects on the image). The models and images are the property of the University of Pecs.

The following algorithm was developed for the automatic evaluation of the peg transfer tests (Fig. 3):

1. The videos were read and split into frames using the OpenCV library.
2. Inference was performed using the trained YOLOv8 model on the frames to determine the type and location of the objects. The capability of object detection is a key element for exercise evaluation, although it does not directly measure task accuracy. Purposing a comprehensive evaluation, a custom algorithm was developed that determined the presence of errors and the overall exercise duration.
3. False positive and false negative detections were filtered out using a sliding average, making the individual states (e.g. grab, drop, rubber–peg contact) significantly more stable.
4. Based on the model results, the exercise time was determined and divided into sessions. A session lasts from the moment a rubber ring is picked up until it is released.
5. The sessions were validated considering the pitfalls. The pitfalls are errors such as missing handover, invalid handover and invalid pickup or release. A missed handover was determined based on the fact that the rubber was grabbed using different instruments at the beginning and the end of the session. By examining the faulty pickup (picked up by the opposite instrument after dropping), an invalid handover could be ruled out—that is, it could be determined that the handover occurred in mid-air.
6. The results were determined from the errors and the measured exercise duration.
7. The results were saved into a csv file for easy comparison.

The algorithm was run on all pre- and post-course videos, and the results were compared with the conventional rating data accessed by the professionals to obtain interrater reliability (professionals’ consensus vs. AI-based algorithm).

For both the standard and AI-based algorithm assessments, the time needed for the evaluation was recorded for every exercise separately. The automatic evaluations were performed on a PC workstation with an Intel^®^ Core^™^ i9-9900KF processor (Intel Corporation, Santa Clara, CA, USA), 3.60 GHz and NVIDIA GeForce RTX 2080 Ti 12 GB graphic card (NVIDIA Corporation, Santa Clara, CA, USA). A graphical processing unit was used for inference.

### Validation of the effectiveness of the developed VR simulator and AI-based automatic assessment software

To validate the effectiveness of the developed VR simulator, the pre- and post-training test results were compared between the VR and control groups. The developed algorithm’s reliability was assessed in several steps. To justify the accuracy of object detection, model evaluation on the test datasets was performed, and the mean average precision metric was determined. The results of the automated evaluation were compared with the experts’ manual assessments to determine accuracy. Through this, it could be verified whether the automated assessment correctly evaluated the exercise or produced false-positive or false-negative results. A false-positive result is when the algorithm incorrectly considers an exercise as passed, and a false-negative result is when the algorithm incorrectly considers an exercise as failed, while the expert evaluator does not.

### Statistical analysis

The data for the pre- and post-course tests were collected from the experts and the algorithm in the same structure. For the analysis of the between-group test results, the chi-squared test was used. To test normality, the Shapiro–Wilks test was performed, and it showed that the examined variables did not follow a normal distribution. Thus, the Mann–Whitney U test was used to compare the independent groups (exercise durations in the VR and control groups). The Wilcoxon signed-rank test was applied to compare the completion times of the study groups. Cohen’s Kappa test was used to determine inter-rater reliability.

Jamovi (version 2; Sydney, Australia) was used for the statistical analysis^40,41^. The significance level was set to p < 0.05. For chart building, Origin (Origin, version number (e.g. ‘Version 2022’); OriginLab Corporation, Northampton, MA, USA.) was used.

### Ethical approval

Based on the current regulations, the University of Pecs Medical School Regional Ethics Committee certified the research to be conducted without ethical authorisation (9749-PTE 2023). Informed consent was obtained from all subjects. All methods were carried out in accordance with relevant regulations. All of the data were collected anonymously, and the study was conducted in accordance with the Helsinki Declaration.

## Results

### VR simulator training effectiveness

**Figure 4:**
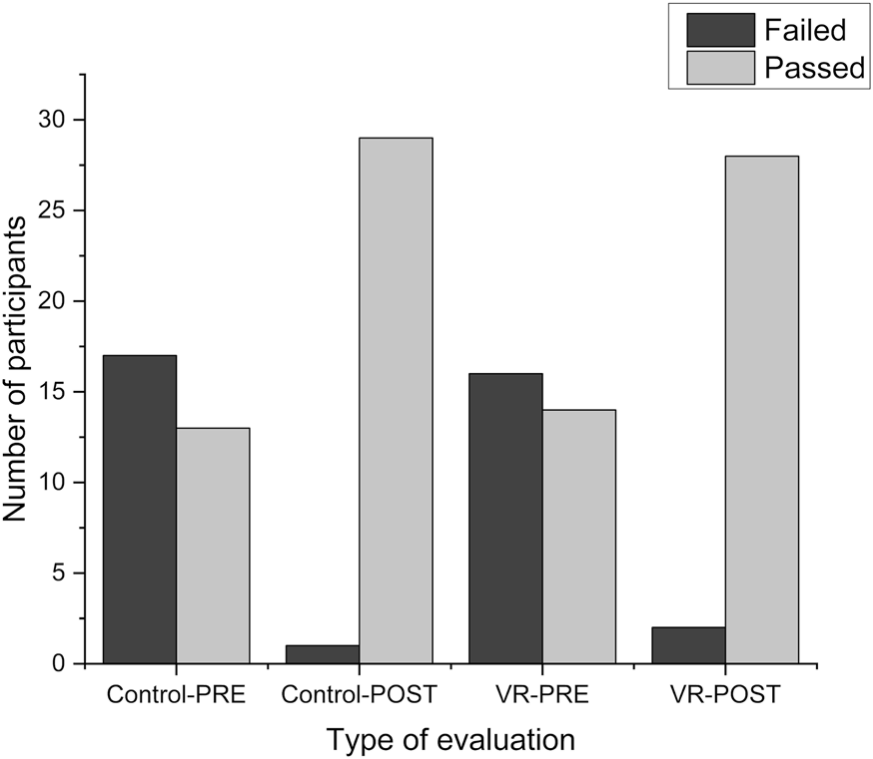
Number of participants who passed and failed the pre- and post-tests in the control and VR groups. The dark grey bars represent the number of ‘Failed’ tests, while the light grey bars show the ‘Passed’ tests. ‘PRE’ refers to the pre-test results, while ‘POST’ refers to the post-test results.

The results of the students who practiced on the VR simulator were compared with the results of the control group. The results of the pre- and post-course tests in the VR and control groups are shown in Fig. 4. More than half of the participants in both groups failed the pre-course test (56.67% in the control group and 53.33% in the VR group), based on the criteria mentioned in Fig. 2. Conversely, during the post-course test, only 3.33% of the students in the control group and 6.66% of those in the VR group failed the test. Based on these post-course test results, the effectiveness of the VR trainer was evaluated and showed no significant difference in the post-course test results irrespective of the practice trainer (VR trainer vs. traditional trainer) using the chi-square test (x^2^ = 0.35, p = 0.55).

To evaluate the individual improvement of the participants based on the applied trainer (VR trainer vs. conventional trainer) during the practice period, the pre- and post-course test results were compared. A total of 46.66% of the students in the VR group and 53.33% of the students in the control group failed the pre-course test and then improved and passed after the training period (for a detailed representation of the changes in outcome, see Supplementary Table 2).

**Figure 5:**
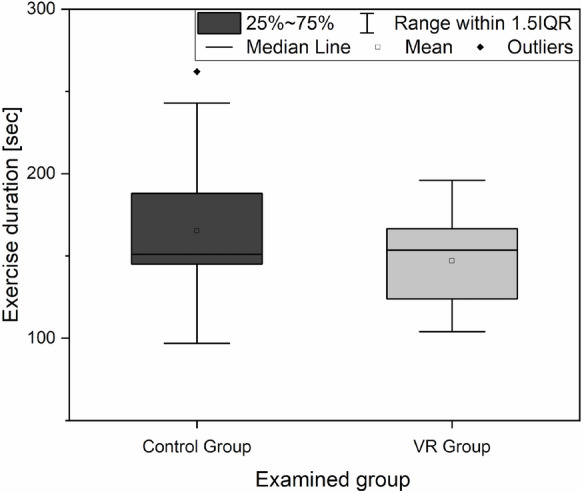
Comparison of exercise duration between the control group and the VR group.

The time needed to perform a given task is a major determinant of the gained laparoscopic skill. Thus, the time needed to fulfil the task during the post-course test was compared between the VR group and the control group, and the results are shown in Fig. 5. No significant difference was found between the two groups in terms of exercise duration based on the Mann–Whitney U test (U = 308, p = 0.118).

To further evaluate the effectiveness of the VR trainer, the improvement of time factor was evaluated in both groups, specifically in the students who passed both the pre- and post-course tests. In the control group, 43.33% of the participants completed the post-course test within a significantly shorter time (average difference: 59 s, Wilcoxon signed-rank: W = 391, p < 0.001). In the VR group, 46.66% of the participants completed the post-course test within a significantly shorter time (average difference: 78 s, Wilcoxon signed-rank: W = 405, p < 0.001), which refers to the substantial improvement in the VR training group compared with the control.

### Feedback from students

**Figure 6:**
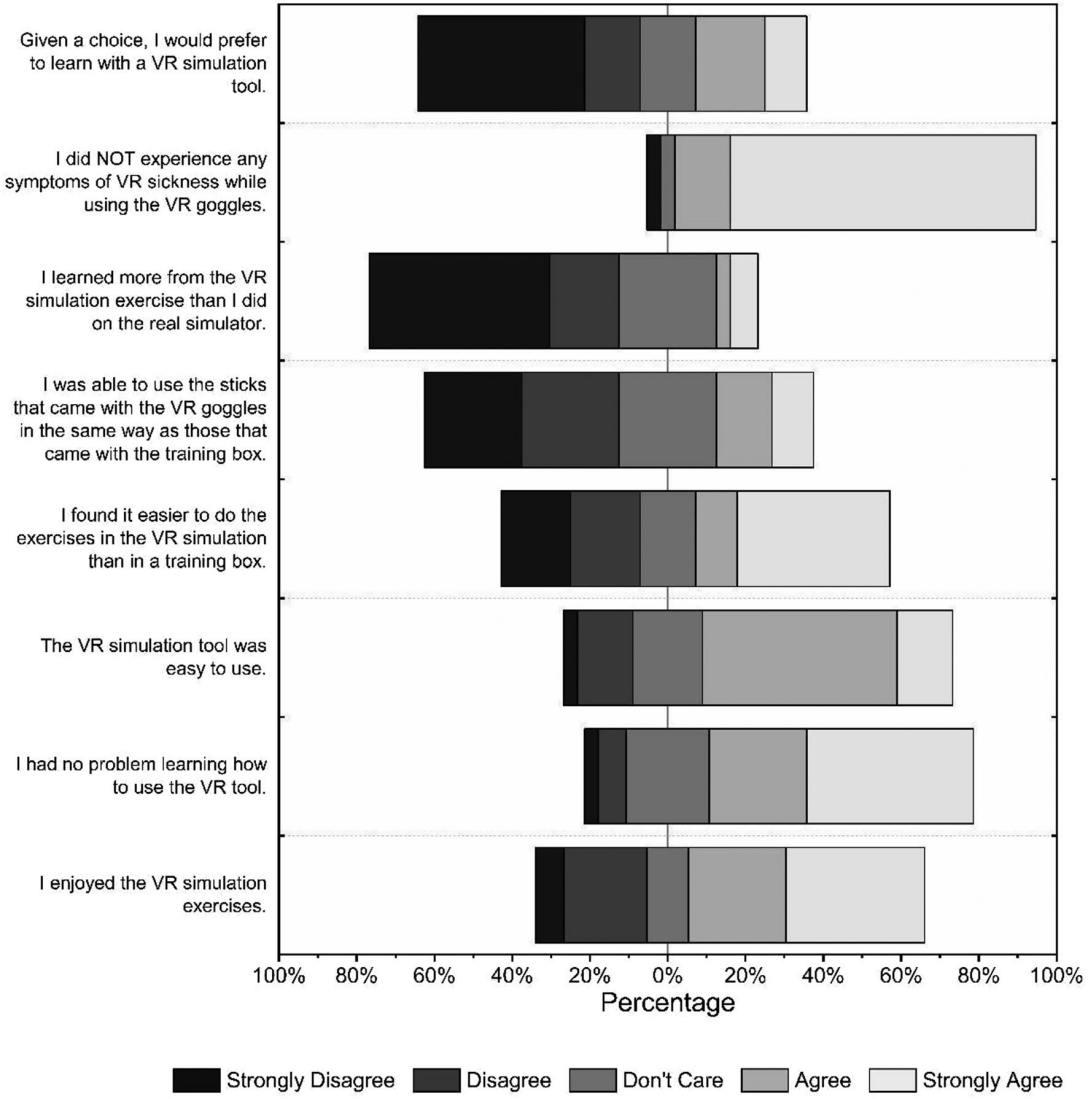
Results of the questionnaire about the VR simulator expressed in percentage of the students’ responses.

To access the subjective user’s experience and impression of the students from the VR training group about the newly developed simulator compared with the traditional FLS trainer used during the pre- and post-course tests, an eight-item Likert scale questionnaire was conducted, and feedback from 88.85% of the participants was obtained. The results of the questionnaire are shown in Fig. 6, while the descriptive statistics are presented in Supplementary Table 1. Based on the responses, the application of the VR trainer and the lack of VR sickness were the most pleasant experiences for the students. The lowest ratings were received for the questions regarding the knowledge gained from the VR simulator and whether the VR simulator was preferred to the traditional trainer.

### Results of the newly developed AI-based evaluation method

To assess the reliability and effectivity of the AI-based evaluation method, the object detection performance, the time measurement and the fail–pass discrimination beyond the overall time needed to perform the complete process were tested and compared with the standard human-based evaluation results. The trained AI models were evaluated using the test datasets, and the object detection performance of the different components involved in the peg transfer exercise was compared (Table 1). The mean average precision is the commonly used metric to determine the performance of object detection, and it is given per object in Table 1.

**Table 1:** Object detection performance metrics: mAP50: mean average precision (mAP) at 0.50 intersection over union (IoU) threshold; mAP50-95: calculates the mAP every IoU thresholds from 0.50 to 0.95 with 0.05 increments then averages the calculated mAP values.

Class	mAP50	mAP50-95
Grab	0.83	0.64
Rubber	0.90	0.63
Dissectors	0.68	0.64
Pegs	0.99	0.66

**Figure 7:**
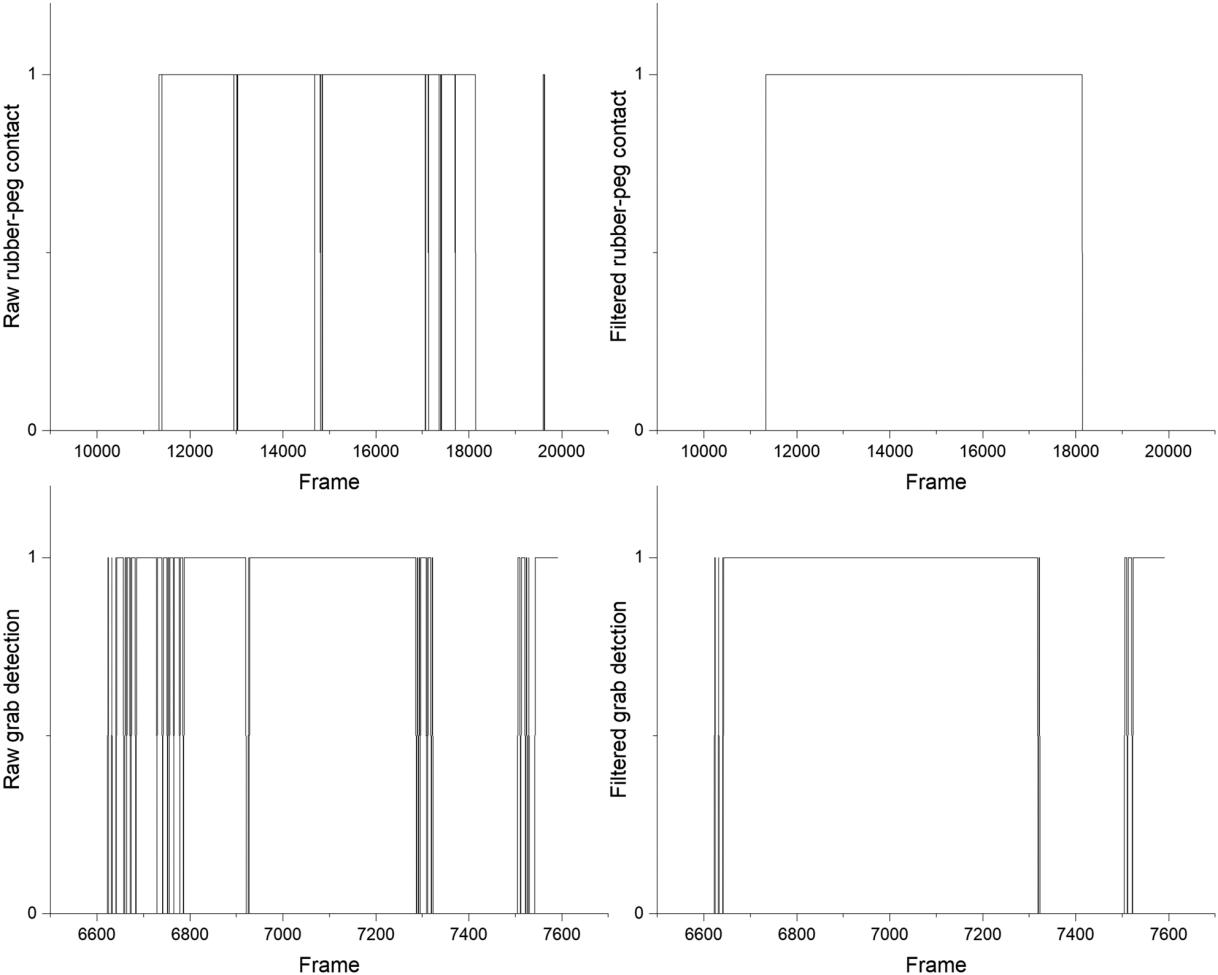
Comparison of raw detections and filter results for grabs and rubber–peg contacts in time segments from a video.

The 240 videos analysed contained 3,038,120 frames, and our algorithm filtered out 301,825 false-negative/positive grab detections and 1,333,781 false-negative/positive rubber–peg contacts. The grabs with up to two possible detections per frame indicate 4.96% error filtering. The rubber–peg contact with up to 12 possible detections per frame represents an error filtering of 3.66%. The error correction effect of the filtered algorithm on the raw detections is illustrated in Fig. 7.

The time needed to perform the tests was evaluated and compared with the standard evaluation values. The average difference between the test time measured by the AI-based algorithm and the standard assessment was 2.61 s, and the standard deviation was 2.93 s. Based on the Mann–Whitney U test, no significant difference was found between the two approaches (U = 28,542.00, p = 0.87).

The duration of the standard manual evaluation and the AI-based evaluation was also compared. The average duration of the manual evaluation was 333 s (the standard deviation was 112 s), and the average duration of the AI-based evaluation was 273 s (the standard deviation was 119 s). The longest evaluation of an exercise was 845 s by the AI-based algorithm and 865 s by the experts. Based on the measured average durations, one expert evaluator could finish all 240 recorded videos in at least 22 h without any breaks; this also indicates the total time required for manual assessment. Conversely, the AI-based algorithm on 10 concurrent threads could finish in around 2 h.

The interrater reliability of the AI and standard human evaluation was assessed based on the preset criteria for passing and failing the test. The evaluation algorithm showed 95% identical results (pass vs. failure) compared with the consensus of the standard human evaluators (Table 2), which was statistically proven by the strong agreement of Cohen’s kappa coefficient (0.90). Conversely, the individual ratings of the experts showed 91.25% agreement, corresponding to a Cohen’s kappa coefficient of 0.82.

**Table 2:** Comparison between AI-based algorithm and standard assessment by the experts.

		AI evaluation	
		Pass	Fail
Standard evaluation by experts	Pass	125	8
	Fail	4	103

## Discussion

This study investigated the effectiveness of laparoscopic training in a VR environment compared with the traditional training box FLS peg transfer exercise. A novel AI-based objective and automated assessment method was developed and validated by comparing it with the standard manual evaluation performed by medical professionals.

The tendency to conduct simulator-based training in graduate and postgraduate medical education has been internationally accepted in recent years^34^. High-fidelity simulators with objective built-in automatic assessment tools require substantial technological background and installation space along with high costs, which limit the availability of these systems^11,12,34^. As potential alternatives, low-fidelity VR simulators, which require less space and investment, have also been implemented, but they have the major drawback of lacking integrated automated objective evaluation systems^9^.

As a substitute, immersive VR solutions can be implemented for simulation-based training methods with suitable self-developed objective evaluation systems^34^.

Several benefits enable immersive VR simulations to be suitable alternatives to traditional training, including pedagogical, technological, practical and economic points. From the educational perspective, immersive VR is engaging and effective in developing fine motor skills, hand–eye coordination and 3D spatial perception; these competencies can be transferred to future clinical work^42–45^. Technological advances are extensive, including eye and hand movement and inside-out tracking or exercise analysis without additional complexity or sensors^44,45^. Immersive VR is a practical solution for home-based use because it is portable and offers an enjoyable user experience in a cost-effective manner^33,34^. A recent review highlighted some of the abovementioned benefits of immersive VR in surgical training. However, it should be noted that these studies only involved a limited number of participants and that there is a clear need to conduct more extensive studies.

The developed immersive VR laparoscopic simulator can be a promising alternative to the traditional box trainer in terms of manual skill development for medical students in undergraduate training, and it has the advantage of automated assessment. The progress of the participants showed no significant difference between the study groups. Furthermore, the exercise duration showed modest improvement in the VR group, which indicates that VR-based FLS training could serve as a reliable and suitable alternative to traditional basic surgical skill education. This observation is especially important, considering the recent and expected further development of distance education approaches in surgical training^13,14^.

In addition to its advantageous training effectiveness, as proven in our research, based on the students’ feedback, the self-developed VR simulator was easy to use and had an enjoyable user experience. The main concern was the dissimilar use of dissectors in the simulator. However, based on the test results, this paucity did not affect the overall efficacy of the simulator but could explain the subjective preference of the traditional trainer.

Although the VR training group achieved similar or better results, considering the students’ feedback about the self-developed VR simulator software, more students subjectively preferred the traditional training box. The students were least satisfied with the divergent haptic perception of the controller compared with the real laparoscopic tool, which could explain their subjective preference for the traditional trainer. Even though the training type did not affect the test results, this feedback about our application was valuable. Therefore, we plan to improve the experience with a more realistic practice environment and easier controller handling. Several studies have reported VR sickness as a general issue, which can manifest as dizziness, nausea or general discomfort and limit education with immersive VR simulators^46,47^. Pathomechanism is multifactorial, including the quality and dynamics of the content and the properties of the used device itself, which should be considered during training material development. Despite the extensive development of head-mounted displays (HMD), which are becoming increasingly available, they still could not solve the problem entirely^48^. Conversely, in the present study, only 6.89% of the students reported mild VR sickness symptoms during the entire training programme, and no students wanted to interrupt their participation because of it. Therefore, any new VR application for educational purposes should be assessed from this perspective before application, as these symptoms may negatively affect learning and encouragement^49,50^.

Based on our results, VR training was found to be feasible and useful for learning surgical skills, and these results are in accordance with previous studies^34^. Qin et al. compared self-developed VR, AR, CVR, MR and box trainer simulators and found that the box trainer was superior in terms of haptic perception, but the other simulators had advantages such as objective evaluation, unlimited reusability and good visual parameters^33^. Although its user experience was positive, the objective evaluation of the exercises and the effectiveness of the training were not compared with the gold standard box trainer^33^. Thus, the self-developed AI-based automatic assessment algorithm specifically targeted the low-fidelity box trainer and was found to be as effective as traditional expert evaluators, with less time needed for the evaluation process. This suggests a future potential to make automated evaluator systems more attractive and widespread. Our study also exceeds the scope of VR validation with the objective analysis of the training efficiency and found the immersive VR training to be as sufficient as the traditional way in a high number of graduate medical school students.

Maciel et al. developed a high-fidelity PC-based laparoscopic VR simulator (VBLaST) for FLS exercises, and subsequently, Chellali et al. validated the VBLaST VR simulator against the FLS box trainer in the peg transfer exercise based on the performance of the participants in the FLS box trainer versus the VBLaST simulator^51,52^. A significantly lower performance was found in the VR simulator, but it was found that the participants improved their performance in both systems, with a significantly greater improvement in the VBLaST simulator^52^. It should be noted that these studies did not use HMD to create an immersive VR environment and did not perform a randomised controlled study design to investigate the effectiveness of the PC-based VR simulator regarding the peg transfer exercise^51,52^. This could explain the difference found in our research, which showed that graduate students practicing in an immersive VR environment were just as successful on the final test as their traditionally trained partners. To expand the reliability of this research, the participants were randomly selected for VR or traditional training methods, and the same training protocol was implemented for both groups (e.g. the participants had no experience with FLS trainers at the beginning of the study and had the same number of practice opportunities).

VR-based simulation has the advantage of having a built-in automatic assessment of participants’ performance. It also saves a significant amount of time for tutors and instructors by avoiding manual assessments. The developed VR software can effectively measure the number of errors and pitfalls and can also detect the pathways of the simulated instrument in real time. However, the analysis of these data is beyond the scope of this study and should be considered in future studies.

Previous research has suggested that AI-based solutions are sufficient to digitalise and automate the analysis and evaluation of simulation-based medical exercises^21–24^. To evaluate basic surgical skills in a simulation-based environment, Zia et al. reported an approach that implemented both the accelerometer and image processing data and found image processing to be superior to the accelerometer in extracting and evaluating skill-relevant information^53,54^. Yanik et al. used a two-stage approach, with the first stage segmenting the movement of the instruments as features from the video and the second stage using these features to assess surgical skills and to determine the task scores without identifying and visualising exercise errors, while our methodology focused on identifying pitfalls to provide human-interpretable objective feedback^55,56^. Yanik et al.’s approach uses AI models without partial outcomes, which are optimised for a fully automated evaluation and act as a black box for the human evaluator^55,56^. By contrast, our approach allows for the visualisation of errors defined as partial outcomes, thereby facilitating human intervention and override. Yamazaki et al. trained an AI model to examine gastrectomy videos by detecting surgical instruments and generated heatmaps^33^. Therefore, the object detection metrics presented in this study verify that image processing could provide objective input data for further evaluation of algorithms in the case of laparoscopic exercises but that these detections are not limited to surgical instruments.

The three major evaluating factors for the FLS success rate are the time needed to perform the exercise and the number of mistakes or pitfalls^18,19^. Belmar et al. developed an AI-based evaluator system that showed promising results in terms of exercise duration measurement (93.02% agreement with the evaluation of experts), but they did not assess the pitfalls^29^. Fathabadi et al. created an evaluator system for both the pitfalls and time measurement, but it required significant modification of the conventional box trainer^31^. Thus, in our study, a novel, AI-based evaluation system was developed not only to measure exercise duration but also to detect mistakes and pitfalls, thereby automating and objectifying the complex evaluation process without modifying the box trainer.

The developed AI-based assessment proved to be as effective and reliable as the human-based standard method in all three fundamental evaluation variables. The average time of evaluation was measured and showed that one exercise evaluation took 333 s using the traditional method and 273 s using the AI-based algorithm. Thus, one evaluator could finish all 240 recorded videos in around 22 h continuously without any breaks, while the AI-based algorithm on 10 concurrent threads could finish in around 2 h. This implies that AI-based evaluation could save instructors a significant amount of time so that they could focus more on other activities necessitating human knowledge, such as teaching or supporting their students, instead of test evaluations.

Regardless of the promising results, this study has some limitations that need to be considered. This study focused only on one FLS exercise. Further developments should cover other FLS skills, such as precision cutting or ligating loops. Based on our initial results, assessments for suturing and knotting are also planned in the next phase, including the current AI-based automated procedure and the VR simulator. For the automatic assessment, an additional side-view video recording should be considered, as the contact between the grabbed rubber and the peg board could not be determined accurately in the current single point of view. However, this requires hardware changes to the original trainer box, which could be a potential drawback. Further experiments involving more experienced participants, such as resident doctors and practicing surgeons, are needed to establish definitively the effectiveness of the VR simulator compared with traditional training tools. Based on the participants’ feedback, we plan to further optimise the VR simulator to enhance the user experience with haptic feedback and to extend our study to postgraduate students and resident doctors.

## Conclusion

In this study, immersive VR-adapted FLS training was developed with a comprehensive AI-based evaluation method. To the best of our knowledge, this is the first study evaluating the peg transfer exercise with an AI-based algorithm, considering not only the duration of the practice but also evaluating the pitfalls, therefore automating and objectifying the complex assessment. The VR simulator is suggested to be feasible for training purposes and is a potentially good alternative to the box training simulator. Based on our results, immersive VR simulators and AI-based assessment algorithms could be used to improve the quality of simulation-based medical education, support independent learning curves and provide objective, standardisable and repeatable assessment methods. These improvements could reduce the burden on trainers and provide more practice opportunities for students, thus improving their manual skills.

### Supplementary Information

Supplementary Tables.

Supplementary Video 1.

## Data Availability

All the annotated data, evaluation sheets and 3D printing files used in the fabrication of the laparoscopic simulator can be found under the following Mendeley Data Repository link: Bogar, Peter; Virag, Mark; Bene, Matyas; Hardi, Peter; Matuz, Andras; Schlegl, Adam; Toth, Luca; Molnar, Ferenc; Nagy, Balint; Rendeki, Szilard; Juhos, Krisztina; Ferencz, Andrea; Fischer, Krisztina; Maroti, Peter (2023), ‘Dataset for the publication entitled “Validation of a novel low-fidelity virtual reality simulator and artificial intelligence assessment approach for peg transfer laparoscopic training”’, Mendeley Data, V1, 10.17632/ncy5btsk4m.1 When citing the dataset, please refer to this article as well.

## Code availability

The developed code is available upon request.
