# Supplementary material for: Validation of a novel, low-fidelity virtual reality simulator and an artificial intelligence assessment approach for peg transfer laparoscopic training
Source: Sci Rep. 2024 Jul 19;14:16702. doi: 10.1038/s41598-024-67435-6 (PMC11271545; doi:10.1038/s41598-024-67435-6)
Supplement: Supplementary file 1 — Supplementary Tables. [file 41598_2024_67435_MOESM1_ESM.docx]

| **Questionnaire item** | **Mean rating** | **Standard deviation** |
| --- | --- | --- |
| 1. Given a choice, I would prefer to learn with a VR simulation tool. | 2.39 | 1.47 |
| 2. I did NOT experience any symptoms of VR sickness (dizziness, nausea, vomiting, headache, malaise, tremors) while using the VR goggles. | 4.64 | 0.87 |
| 3. I learned more from the VR simulation exercise than I did on the real simulator. | 2.07 | 1.25 |
| 4. I was able to use the sticks that came with the VR goggles in the same way as those that came with the training box. | 2.61 | 1.32 |
| 5. I found it easier to do the exercises in the VR simulation than in a training box. | 3.36 | 1.59 |
| 6. The VR simulation tool was easy to use. | 3.57 | 1.03 |
| 7. I had no problem learning how to use the VR tool. | 3.96 | 1.14 |
| 8. I enjoyed the VR simulation exercises. | 3.61 | 1.37 |

**Supplementary Table 1**: Descriptive statistics of the Likert scale about the VR simulator

| **Outcome** | **Control group** | **VR group** |
| --- | --- | --- |
| F-F | 1 (3.33%) | 2 (6.66%) |
| P-P | 13 (43.33%) | 14 (46.66%) |
| F-P | 16 (53.33%) | 14 (46.66%) |
| P-F | 0 (0%) | 0 (0%) |

**Supplementary Table 2**: Comparing the progress of the students results based on the pre- and post- course tests pass or failure rate in the two practice groups, F-F=failed both, P-P=passed both, F-P=failed pre, passed post, P-F=passed pre, failed post
